# Papillary Thyroid Cancer in a Child with Progressive Transformation of Germinal Centers

**DOI:** 10.1155/2016/6469073

**Published:** 2016-03-16

**Authors:** Suresh Mohan, Bradley DeNardo, Dariusz Stachurski, Jennifer Greene Welch, Jan C. Groblewski

**Affiliations:** ^1^The Warren Alpert Medical School of Brown University, Providence, RI 02903, USA; ^2^Division of Pediatric Hematology/Oncology, Hasbro Children's Hospital, Providence, RI 02903, USA; ^3^Department of Pathology, Rhode Island Hospital, Providence, RI 02903, USA; ^4^Division of Otolaryngology, Hasbro Children's Hospital, Providence, RI 02903, USA

## Abstract

*Objectives*. To describe the presentation and management of a child with Progressive Transformation of Germinal Centers (PTGC), an uncommon condition characterized by significant persistent lymphadenopathy, who developed papillary thyroid carcinoma and to explore and review potential links between PTGC and neoplastic processes in the head and neck.* Methods*. Case presentation and literature review are used.* Results*. A 10-year-old female presented with a right parotid mass and cervical lymphadenopathy. Multiple biopsies revealed PTGC without malignancy. Two years later, she developed fatigue and weight gain, and a thyroid nodule was found. Fine needle aspiration was strongly suggestive of papillary thyroid carcinoma. The patient underwent total thyroidectomy and central neck dissection without surgical management of the longstanding right lateral neck lymphadenopathy. Final pathology confirmed papillary thyroid carcinoma. She was treated with radioactive iodine therapy postoperatively and remains free of disease at three years of follow-up.* Conclusions*. PTGC is considered a benign condition but has previously been associated with Nodular Lymphocyte Predominant Hodgkin Lymphoma (NLPHL). This is the first reported case of papillary thyroid cancer in a child with preexisting cervical PTGC and no defined risk factors for thyroid malignancy. No link has been established with thyroid carcinoma, but patients with PTGC may have a defect in immune surveillance that predisposes them to malignancy.

## 1. Introduction

Progressive Transformation of Germinal Centers (PTGC) is an uncommon condition characterized by significant persistent or recurrent lymphadenopathy [1, 2]. The etiology of PTGC is unknown, but it has been proposed to be the result of abnormal follicular hyperplasia following antigenic stimulation [3]. Associations with autoimmune conditions have also been proposed [4]. PTGC has a male predominance (3 : 1) and is 4 times more common in the adult population than in the pediatric population [1, 2, 5]. Cervical lymph nodes are involved in approximately 50% of cases, but other common sites for PTGC include inguinal and axillary lymph nodes [1, 4]. Single node involvement is more common than multiple node involvement [1]. Involved lymph nodes typically contain germinal centers that are three to five times larger than typical reactive follicles [2]. Histologically, PTGC can be difficult to distinguish from Nodular Lymphocyte Predominant Hodgkin Lymphoma (NLPHL) [1, 4]. Nodes typically demonstrate expansion of the small mantle zone lymphocytes into adjacent sinusoids and into the germinal centers, effectively obliterating the distinction between the germinal center and the mantle zone [1]. PTGC has been previously implicated in the development of NLP Hodgkin lymphoma, yet this association is controversial and the clinical implications of this diagnosis remain unclear.

Thyroid cancer is an uncommon diagnosis in childhood. Over 90% of childhood thyroid cancers are papillary thyroid carcinoma or its follicular variant [6]. Papillary thyroid cancer is very rare in children, accounting for only 1.4% of all pediatric malignancies with a peak incidence between 15 and 19 years of age [7]. Although the most well described risk factor for papillary thyroid cancer is a history of ionizing radiation, only 3% of current childhood cases have a history of radiation exposure to the head or neck. Other conditions predisposing to papillary thyroid cancer include adenomatous polyposis coli and Cowden syndrome. Familial cases of childhood papillary thyroid cancer occur in only 3%. The majority of cases of childhood papillary thyroid cancer are sporadic in nature with children typically presenting with a palpable thyroid nodule and no associated symptoms. These patients achieve an excellent overall prognosis with appropriate multimodal therapy including total or near-total thyroidectomy and lymph node dissection as well as radioiodine remnant ablation and thyroid hormone suppressive therapy to achieve a disease-free status [6, 7]. Here, we report a case of this rare pediatric malignancy occurring in the setting of known PTGC and persistent cervical lymphadenopathy.

## 2. Case Report

**Figure 1:**
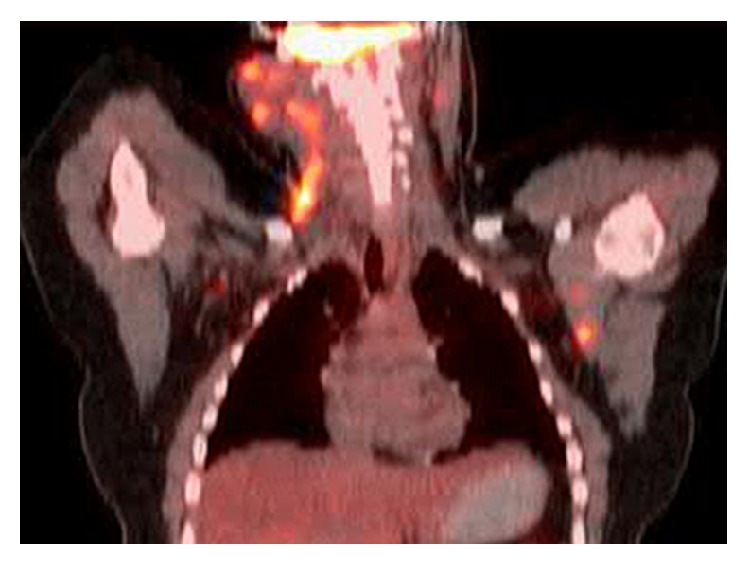
Positron Emission Tomography, Computed Tomography Scan. Fusion PET/CT demonstrating diffuse right cervical and left axillary lymphadenopathy.

**Figure 2:**
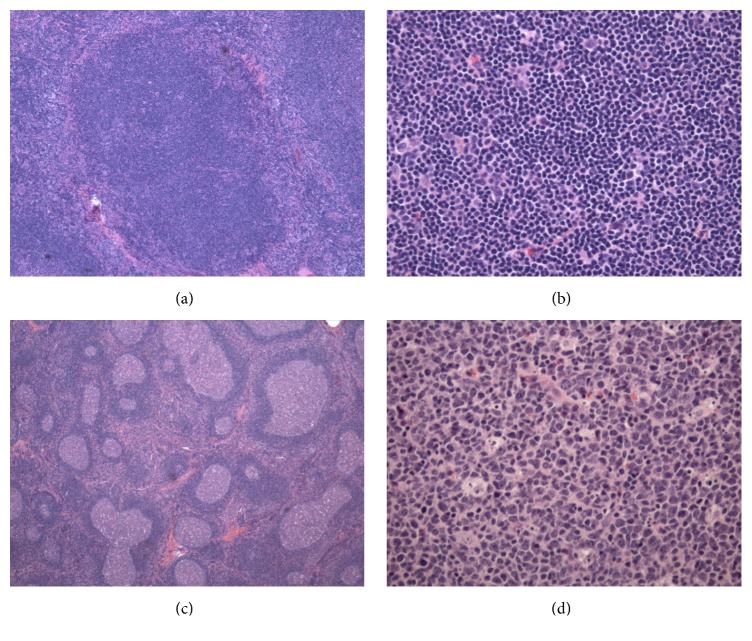
PTGC microscopic examination. (a) PTGC, markedly enlarged lymphoid follicle infiltrated by small mature mantle zone lymphocytes with gradual replacement of germinal center (H&E, 4x). (b) Background of florid follicular lymphoid hyperplasia (H&E, 2x). (c) PTGC, germinal center replaced by small mature mantle zone lymphocytes (H&E, 20x). (d) Reactive germinal center with numerous tingle body macrophages (H&E, 20x).

**Figure 3:**
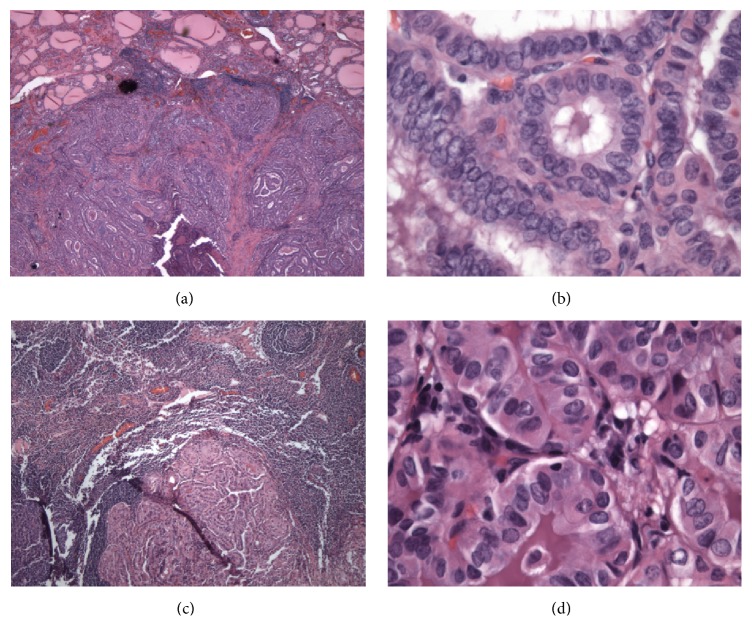
Papillary thyroid cancer microscopic examination. (a) Papillary thyroid carcinoma. Circumscribed nodule with papillary and follicular architecture and background fibrous bands (H&E, 2x). (b) Nuclear crowding, oval to elongated nuclei with fine open chromatin, nuclear membrane irregularity, and nuclear grooves (H&E, 40x). (c) Lymph node showing metastatic papillary thyroid cancer (H&E, 4x). (d) Metastatic papillary thyroid cancer nuclear features (nuclear crowding, fine open chromatin pattern, nuclear grooves, and nuclear membrane irregularity) (H&E, 40x).

A 10-year-old female with a one-year history of a progressively enlarging right parotid mass was referred for pediatric otolaryngology evaluation. The patient had no known risk factors for head and neck malignancies. There was no family history of thyroid or other malignancies and no history of childhood radiation. Examination revealed a 2 cm right parotid mass with associated cervical lymphadenopathy. Workup for autoimmune disease revealed CBC, ESR, ANA, and immunoglobulin tests to be within normal limits with no immunophenotypic evidence of ALPS. CT demonstrated enlarged right intraparotid and cervical lymph nodes. Ultrasound-guided biopsy of the parotid mass revealed a reactive lymphoid process. Excisional biopsy of the multiple cervical lymph nodes revealed florid follicular lymphoid hyperplasia with few follicles demonstrating infiltration of the germinal center by small mature mantle zone lymphocytes consistent with a pathologic diagnosis of PTGC. Due to the potential association with NLPHL, PET/CT was performed, and increased uptake was noted in the right parotid, bilateral jugulodigastric chains, right supraclavicular region, right axilla, peripancreatic region, and bilateral inguinal regions (Figure 1). Additional excisional biopsy of a particularly high FDG uptake supraclavicular lymph node revealed PTGC without any evidence of malignancy (Figure 2). The patient was followed up clinically and the lymphadenopathy remained stable for two years. Incidentally, the patient was evaluated by her primary care physician for thyroid abnormalities after developing unexpected weight gain and fatigue. Laboratory workup was negative for hypothyroidism. Thyroid ultrasound demonstrated an isolated left thyroid nodule and subsequent FNA was suggestive of papillary thyroid carcinoma. Total thyroidectomy with central neck dissection was performed. Despite the presence of persistent bilateral jugulodigastric lymphadenopathy, the lateral necks were not addressed surgically. Pathology confirmed papillary thyroid carcinoma (Figure 3), and two of four lymph nodes demonstrated metastatic disease without extranodal invasion. Pathologic TNM stage was T1a, N1a, and M0 (Stage 1). No lymph nodes had evidence of PTGC. Postoperative radioactive iodine scan showed minimal uptake in the thyroid bed and no uptake in the lateral necks. The patient received adjunct radioiodine (RAI) ablation therapy. The patient remains well without evidence of recurrent disease 3 years after treatment.

## 3. Discussion

First described in 1975, PTGC is a rare clinicopathological entity that results in chronic lymphadenopathy. PTGC is considered a reactive process that usually occurs in the setting of follicular hyperplasia [1, 2]. Histologically, PTGC generally occurs as well-defined macronodules scattered in the background of typical follicular hyperplasia, which are usually at least twice as large as the hyperplastic follicles. In most cases, single or a few transformed germinal centers are present in a lymph node. Epithelioid histiocytes may occasionally be seen surrounding the follicles but not within follicles such as what may be encountered in NLPHL. Per immunohistochemistry, the small cells are predominantly IgM^+^, IgD^+^ mantle zone B cells. CD21^+^, CD23^+^ follicular dendritic meshworks highlight the intact follicles. The main differential diagnosis is nodular lymphocyte predominant Hodgkin's lymphoma (NLPHL). NLPHL also histologically presents as macronodules, usually with irregular or indistinct borders, which efface the nodal architecture and lack interspersed reactive follicles. The nodules are composed of predominantly small B cells with scattered large cells, which represent Reed-Sternberg variants known as “popcorn cells” or LP cells. An immunohistochemical characteristic in NLPHL not seen in PTGC is the rosetting of CD57^+^ T-cells around the neoplastic CD20^+^ LP cells.

Patients with PTGC typically present with persistent or recurrent nonspecific lymph node enlargement without associated symptoms. Superficial lymph node sites are most commonly involved, with head and neck involvement accounting for 50% of cases. The exact incidence of PTGC is unknown, although previous studies have demonstrated PTGC in 4-5% of biopsies performed for chronic lymphadenopathy [2, 8]. It has been suggested that PTGC incidence is underestimated due to underreporting in pathology specimens in which the PTGC component is negligible compared to an otherwise unremarkable lymph node. In addition, recurrent lymphadenopathy secondary to PTGC resulting in serial lymph node biopsies is well described [4, 5, 8]. As such, PTGC is increasingly recognized as a diagnostic consideration in the evaluation of chronic lymphadenopathy, particularly in the head and neck region.

Despite increasing recognition of PTGC, the clinical significance of this diagnosis remains unclear. Although it is considered a reactive process, PTGC also demonstrates the ability to both grow and recur [2, 3, 9]. PTGC was first proposed as a premalignant lesion in 1979, although the malignant potential of PTGC remains controversial [10]. There is a well-documented association between PTGC and HL, specifically NLPHL, and less commonly with classical HL [3, 5, 8]. Pathologically, PTGC can be difficult to distinguish from NLPHL and may be found concurrently on nodal biopsy. PTGC is reported to occur both before and after a diagnosis of HL as well. PTGC is well described as a common cause of recurrent lymphadenopathy in patients previously treated for HL, with an incidence reported as 1–15% [8, 9, 11]. Previous reports suggest that persistent PTGC significantly increases the risk for development of HL or may be a precursor to relapsed HL after initial treatment [1, 12]. Given this concern for malignant transformation of PTGC, Picardi et al. prophylactically treated a cohort of 48 patients in complete remission from HL who subsequently developed PTGC after therapy with rituximab, an anti-CD20 monoclonal antibody [12]. When compared to a historical cohort of patients with PTGC post-HL therapy, they found a significantly decreased recurrence rate of HL.

PTGC has been associated with hematologic malignancies other than HL including multiple myeloma and low grade follicular lymphoma [3]. Our report is the first to demonstrate an association between PTGC and a nonhematologic malignancy. Recently, PTGC has been found to be associated with multiple nonmalignant disorders as well. Shaikh et al. published a retrospective review of all cases of PTGC diagnosed at a single institution [4]. Of the 29 patients identified, 7 had PTGC associated with an autoimmune disorder, including lupus, immune thrombocytopenic purpura (ITP), Castleman's disease, and a probable case of autoimmune lymphoproliferative syndrome (ALPS). Two additional studies have demonstrated an association between PTGC and IgG4-related disease, which is a recently described lymphoproliferative disorder characterized by tumor-like infiltration of organs with lymphoplasmacytic IgG4-positive plasma cells [13]. IgG4-related disease typically responds to therapy with corticosteroids.

Given the association of PTGC with lymphoma, autoimmune disease, IgG4-related disease, and our case of thyroid malignancy, the authors speculate that PTGC is indicative of an underlying defect in immune surveillance. Defective immune surveillance could result in the accumulation of genetic mutations that predispose PTGC patients to malignant transformation. This concept is supported by the work of Bouron-Dal Soglio et al. who identified a* BCL6* gene translocation commonly found in lymphomas in a 12-year-old male with isolated PTGC [14], suggesting that this mutation occurred as a primary event and that “second hit” mutations are required for malignant transformation. We theorize that our patient has a defective immune surveillance mechanism leading to recurrent lymphadenopathy with PTGC and predisposing to secondary malignancy. Given the rare nature of PTGC, as well as the exceedingly low incidence of papillary thyroid cancer in this age group, the authors speculate that the coexistence of PTGC and papillary thyroid cancer in this patient is unlikely to have occurred by chance alone. Nevertheless, the association between these diagnoses will only be substantiated with serial observations of the same occurrence in additional patients.

## 4. Conclusion

The management of patients with isolated PTGC remains challenging. In addition, the association of PTGC with secondary malignancy as well as autoimmune conditions is unclear. Close surveillance of such patients is necessary. Unfortunately, patients with PTGC are subject to recurrent lymph node biopsies given concern for malignancy [4]. The decision to perform repeat lymph node biopsies must be made on an individualized basis. Increased awareness of PTGC and better understanding of its potential association with autoimmune disease and secondary malignancy are needed.
